# Role of human recombinant activated protein C and low dose corticosteroid therapy in sepsis

**DOI:** 10.4103/0019-5049.72637

**Published:** 2010

**Authors:** Aparna Shukla, Shilpi Awasthi

**Affiliations:** Department of Anaesthesia and Critical Care, ERA’S Lucknow Medical College, Lucknow, India

**Keywords:** Corticosteroid, human recombinant activated protein C (rhAPC), sepsis

## Abstract

Despite advances in modern medicine, sepsis remains a complex syndrome that has been associated with significant morbidity and mortality. Multiple organ failure associated with sepsis leads to high mortality and morbidity. About 28 – 50% deaths have been reported in patients with sepsis. The number of sepsis patients is increasing, with considerable burden on healthcare facilities. Various factors leading to a rise in the incidence of sepsis are (1) Improvement of diagnostic procedures (2) Increase in the number of immunocompromised patients taking treatment for various autoimmune disease, carcinomas, organ transplantation (3) Advances in intensive procedures (4) Nosocomial infections (5) Extensive use of antibiotics. With the better understanding of sepsis various modalities to modify pathophysiological response of septic patients have developed. Activated protein C and low-dose corticosteroid therapy have been tried in patients, with variable results.

## INTRODUCTION

Sepsis syndrome, a systemic response to infection, can produce devastating outcomes even in previously normal individuals. Advances in the treatment of sepsis have led to an attempt to elucidate the ideal plan of management in septic patients. Some investigators advocated the use of low-dose corticosteroid therapy,[1] while others voted for Activated protein C, as soon as the patient arrived in the hospital.[2] In the past, some researchers have also used high-dose corticosteroids to suppress the inflammatory response in sepsis, but it was not found to be beneficial.[3] Although in the past few decades, with advancement in medical science, the outcome of septic patients has improved, yet a controversy exists regarding the use of some treatment modalities like Activated protein C and low-dose corticosteroid therapy. Here, in this article, we have discussed a brief pathophysiology of sepsis, the role of these two agents in sepsis management and various research work done on these two agents till the recent past.

## DEFINITION OF SEPSIS AND SEVERE SEPSIS

In 1992, the American College of Chest Physician / Society of Critical Care Medicine have defined sepsis under the following heads:[4]

SIRS (Systemic inflammatory response syndrome): Altered pathophysiology without positive blood culture.

Sepsis: SIRS induced by infection.

Severe sepsis: Sepsis with dysfunction of at least one organ or organ system.

Septic shock: Severe sepsis with hypotension.

In 2001, the International Sepsis Definition Conference developed another staging system, designated by the acronym PIRO.[5]

*P*_ Pre-existing co-morbid conditions that will reduce survival.

I_Insult or infection.

R_Response to infectious challenge.

O_Organ dysfunction or organ failure

## PATHOPHYSIOLOGY OF SEPSIS

Normal haemostasis exists as a finely tuned balance between the coagulation cascade and fibrinolysis, where the blood typically remains in the liquid condition to allow free flow of blood in the vessels, yet clots appropriately to control bleeding. In sepsis there is total disruption of this fine tuning, shifting the balance toward increased coagulation. Endotoxins released from the cell wall of gram negative bacteria cause the activation of factor X, generation of thrombin (factor IIA) and deposition of fibrin (clot) in the microvasculature. Akasa and others,[6] injected endotoxin in rats and they observed microthombi in hepatic circulation within five minutes. When continuously exposed to endotoxin, there is a deposition of fibrin clots through the microvasculature of the body, resulting in focal areas of hypoperfusion and tissue necrosis thus causing multiple organ dysfunctions.

In normal circumstances whenever there is excessive coagulation, the tissue plasminogen activator generates plasmin from plasminogen, which then lyses the fibrin clots. In sepsis this compensatory mechanism is impaired. Inflammatory cytokines and the thrombin released in sepsis cause inhibition of fibrinolytic enzymes in two ways. First, they activate the platelet and endothelium, to release the plasminogen activator inhibitor; second, the thrombin activates the thrombin activatable fibrinolysis inhibitor. Both these factors inhibit the formation and activation of plasmin and thus impair the fibrinolytic system.[7]

In normal patients, the formation of thrombin is regulated by the anticoagulant system in the body like protein C, antithrombin and the tissue factor pathway inhibitor. Protein C is converted into activated protein C by the thrombin-thrombomodulin complex, an endothelial cell surface receptor.[8] Activated protein C then inactivates factor Va and factor VIIIa, which are the key factors in the formation of thrombin. However, in sepsis, due to endothelial injury the level of thrombomodulin on the endothelial surface decreases, thus conversion of protein C to activated protein C is also reduced.[9–11]

## MANAGEMENT OF SEVERE SEPSIS

### A. Standard therapy

1. Initial resuscitation: Aggressive fluid resuscitation with either natural / artificial colloids or crystalloids is required during the first six hours. The goals of initial resuscitation of sepsis-induced hypoperfusion should include — (1) Central venous pressure 8–12 mm Hg (2) Mean arterial pressure (MAP)≥65 mm Hg.
2. Early diagnosis and antibiotic treatment: Intravenous antibiotic therapy should be started as early as possible. Appropriate cultures should be obtained before antimicrobial therapy is initiated, if such cultures do not cause significant delay in antibiotic administration. Specific anatomical diagnosis of infection requiring consideration for emergent source control should be sought and diagnosed or excluded as rapidly as possible.
3. Treatment of underlying cause: It may require surgical intervention, such as drainage of abscess, laparotomy and so on.
4. Vasopressors: Norepinephrine or dopamine should be used as first choice vasopressor agents to correct hypotension in septic shock. Low-dose dopamine is not recommended for renal protection.
5. Mechanical ventilation: Invasive or noninvasive ventilatory support may be required in patients in whom pulmonary functions are compromised and they are not able to maintain optimal oxygen saturation.

### B. Therapy directed to revert / inhibit pahophysiological response in sepsis

- Human recombinant activated protein C
- Low-dose corticosteroid therapy

### Human recombinant activated protein C (rh APC)

Food and drug association has approved only one drug till date and that is the human recombinant activated protein C (Drotrecogin alfa), for therapeutic interventions in patients with sepsis.

### Pharmacokinetics

Protein C, a major physiological anticoagulant is an endogenous protein in humans, encoded by the PROC gene, with the ability to modulate both inflammation and coagulation. Protein C is made in the liver and circulates as a plasma zymogen (an inactive precursor of protease). This vitamin K dependent serine protease enzyme is activated to become APC on the endothelial surface by the thrombin-thrombomodulin complex.[12] Activated Protein C demonstrates a biphasic half life (t1/2) with a t1/2a and t1/2 b of 13 minutes and 1.63 hours, respectively. Activated Protein C is inactivated by endogenous plasma protease inhibitors. Due to the short half-life and metabolism, rapid elimination of the drug occurs after stopping the infusion. The volume of distribution (Vd) is comparable to the extracellular volume in healthy adults (16 – 20 L).

Chemical formula: C_1786_ H_2779_ N_509_ O_519_ S_29M_

Molecular Weight: 55,000 gram/mol

Bioavailability: 100% (Intravenous application only)

Metabolism: Endogenous plasma protease inhibitor

Half-life: Less than 2 hours.

### Pharmacodynamics

Activated protein C has three mechanisms of action:

1. Acts as an anti-inflammatory agent: It exerts an anti-inflammatory effect through indirect inhibition of Tumour Necrosis Factor Alfa, by blocking the leucocyte adhesion to selectin and by limiting thrombin-induced inflammatory responses within the vascular endothelium.
2. As an anticoagulant agent: Through inhibiting factor Va and factor VIIIa, thus, limiting the thrombin formation.
3. As a pro-fibrinolytic agent: Through inhibition of the plasminogen activator inhibitor-1 and decreased activation of the thrombin activatable fibrinolysis inhibitor.

### Dosage regimen of activated protein C

Drotrecogin alfa is a lyophilised powder that must be reconstituted prior to dilution. It is stable in 0.9% Normal saline (NS) at a concentration of 100 – 200 mcg/ml. It must be administered through a dedicated intravenous line. It can be administered concurrently with 0.9% normal saline, Ringer’s lactate, or dextrose solution through the same line.[13] It is given in multiple infusions in a total duration of 96 hours, provided the maximum duration of one infusion is not more than 12 hours. The dosage is calculated using the formula:

Mg of drotrecogin=patient weight in kg×24 mcg / kg /hour×(hours of infusion)/1000

## ADVERSE EFFECTS AND CONTRAINDICATIONS

Being a potent anticoagulant, the major adverse effect is an increased risk of bleeding and therefore rhAPC is contraindicated in patients with an increased risk of bleeding. The contraindications are:

- Active internal bleeding
- Recent haemorrhagic shock (within three months)
- Recent intra-cranial or intra-spinal surgery or severe head trauma (within two months)
- Trauma with increased risk of life threatening bleeding
- Presence of epidural catheter
- Intra-cranial neoplasm or mass lesion or evidence of cerebral herniation

## MONITORING PARAMETERS

If there is any evidence of bleeding, periodic haemoglobin, haematocrit, coagulation profile and complete blood picture should be done. As drotrecogin alfa prolongs APTT, it is not a reliable marker of the coagulation profile.

## PRECAUTIONS

- For percutaneous procedures, the drug should be stopped two hours prior to the procedure and can be started after one hour.
- For elective surgical procedures, the drug should be withheld 12 hours before and 12 hours after the procedure.
- Drug should be stopped at least two hours before the emergency procedure.
- All patients on Drotrecogin alfa should receive stress ulcer prophylaxis, such as histamine-2 antagonists.
- For uncomplicated, less invasive procedures, the drug can be restarted immediately.
- If a patient requires full dose therapeutic heparin or there is evidence of active bleeding, the drug should be stopped immediately.

## DRUG INTERACTIONS

Human recombinant activated protein C should be used cautiously with other drugs that effect haemostasis concomitantly, such as, aspirin, warfarin and clopidogril; low-dose prophylactic heparin therapy can be given concurrently with drotrecogin.

## STUDIES ON ACTIVATED PROTEIN C

Several studies have reported that the level of APC is low in septic patients and these levels predict the outcome.[14,15] Taylor and others[16] conducted a study of gram-negative septicaemia in baboons; administration of APC along with a 100% lethal dose of *E. coli* prevented the lethality in all the animals tested. When the animals were pre-treated with an antibody specific for activated protein C, the injection of a sub-lethal dose of the organism became 100% lethal.

In July 1998, a multi-center, randomised, double blind, placebo-controlled trial of 1690 patients with severe sepsis was conducted. The trial, which was completed in 2001, is popularly known as the PROWESS (recombinant human activated protein C worldwide evaluation in severe sepsis) trial.[17] Patients either received drotrecogin alfa at the rate of 24 mcg /kg/hour or placebo for 96 hours of total infusion time. APACHE II (Acute Physiology and Chronic Health Evaluation) was calculated during the 24-hour period, immediately preceding the start of drug administration. Statistical analysis indicated that the 28-day mortality was 30.8% in the placebo group and 24.7% in the drotrecogin alfa group. Thus, there was an absolute risk reduction in the mortality of 6.1%The difference in the mortality between patients given APC and those given placebo was limited to patients with high risk of death, that is, APACHE II scores ≥25. In these groups mortality was reduced from 44% in the placebo group to 31% in the treatment group. However, the efficacy was doubtful in patients with low risk of death (APACHE II<25). Serious bleeding occurred more often in patients receiving drotrecogin alfa (3.5%) than in patients receiving placebo (2%), in the PROWESS trial. The results clearly indicated that one in every five patients who would have died was saved with drotrecogin alfa treatment..

In 2002 the European Medicine Evaluation Agency (EMEA) approved the use of APC in patients with severe sepsis and multiple organ failure with an annual required review.[18]

In 2005, the ADDRESS trial submitted its report. This trial, required by the FDA, evaluated the standard 24 mcg/kg dose of APC for 96 hours in a double blind, placebo-controlled, multi-center trial, in patients with severe sepsis and APACHE II score<25 or in patients with single organ failure. No significant difference was found in the 28-day mortality (17% placebo versus 18.5% APC, *P*=0.34). The rate of serious bleeding was 2.4% with APC and 1.2% with placebo, during the infusion period.[19]

The RESOLVE trial with 240 children getting APC and 237 children getting placebo submitted its report in 2007.[20] In this trial too, the 28-day mortality rate was not improved significantly (Placebo 17.5% versus APC 17.7%, *P*=0.39). Unlike the previous studies, the risk of bleeding was equal for placebo and APC (6.8% placebo and 6.7% APC, *P*=0.97).

The most recent meta-analysis on APC from the Cochrane Database submitted its review in 2008. The review included 4434 adults and 477 paediatric patients and did not find any significant reduction in the 28-day mortality in adults. However, the risk of bleeding was increased.[21]

On recommendation of FDA another multi-center, placebo-controlled trial has been started to determine the efficacy of APC, called the PROWESS-SHOCK trial. The trial is expected to be completed by the end of 2011.[22]

After the PROWESS trial, investigators were very enthusiastic about use of APC in all septic patients. Now its use is restricted to selected patients with severe sepsis because of two reasons, (1) cost of treatment with APC is very high, and (2) reduction in the 28-day mortality is not very significant in each and every septic patient.

On the basis of these studies, the SSC guidelines suggested that adult patients with sepsis-induced organ dysfunction associated with a clinical assessment of high risk of death, most of whom will have APACHE II≥25 or multiple organ failure, should receive rhAPC if there are no contraindications (grade 2B except for patients within 30 days of surgery, for whom it will be grade 2C). The SSC guidelines further recommended that adult patients with severe sepsis and low risk of death, most of whom will have APACHE II<20 or one organ failure, should not be given rhAPC (grade 1A). The effects in patients with more than one organ failure, but APACHE II<25 are unclear, and in such circumstances one may use the clinical assessment of the risk of death and the number of organ failures, to support the decision.

## LOW-DOSE CORTICOSTEROID THERAPY IN SEPSIS

In sepsis there is an imbalance between pro-inflammatory and anti-inflammatory cytokines. There is an increase in the concentration of factors IL-1, IL-6 and TNF-α, which are released in abundance from the lymphocytes, macrophages and endothelial cells, during the development of sepsis.[23] Studies show that NF-kappa B activity was highest in the non-survivors of sepsis.[24,25]

Corticosteroids could reduce the exaggerated inflammatory response in sepsis and prove beneficial by,

1. Inhibiting the pro-inflammatory, cytokine-like factor NF-kB, both directly and indirectly.[26]
2. Promoting the production of anti-inflammatory cytokines such as IL-4 and IL-10. Several studies have suggested that the non-survivors of sepsis have a lower concentration of anti-inflammatory cytokines[27,28] and a higher concentration of pro-inflammatory cytokines.[29–31]
3. Enhancing the activity of adrenergic receptors.
4. Increasing myocardial contractility.
5. Corticosteroids inhibit inducible nitric oxide synthetase, a vasodilator molecule.[32] They also inhibit serum phospholipase A2, resulting in decreased production of vasodilator PG E and prostacycline.[33] Thus, the overall effect is an increase in blood pressure.[34]

### Adrenal insufficiency in sepsis

Relative adrenal insufficiency and resistance to glucocorticoid may arise during severe sepsis. TNF-alfa and IL-6 decrease cortisol production from the adrenal gland and ACTH production from the pituitary gland.[35,36] Average production of cortisol by the adrenal gland is approximately 5.7 mg/m^2^ of the body surface per day. In conditions of sepsis, the adrenal gland may produce 150 – 200 mg/m^2^ of cortisol daily. If the adrenal glands are exposed to continuous activation by pro-inflammatory cytokines, the glands are exhausted, thereby causing relative or absolute adrenal insufficiency. Relative adrenal insufficiency is more common, seen in 16.3 to 55% of the patients with septic shock. Absolute adrenal insufficiency is seen in only 3% of the patients.[37,38]

### Selection and dosages of steroid

The most preferred corticosteroid in sepsis is Hydrocortisone. It is usually given 200 – 300 mg/dl in divided doses or in a continuous infusion. Hydrocortisone is preferred because it is the synthetic equivalent to the physiological final active cortisol. Therefore, treatment with hydrocortisone directly replaces the cortisol independent of metabolic transformation. Another advantage of hydrocortisone is that it has intrinsic mineralocorticoid activity. Annane D[39] used fludrocortisone in addition to hydrocortisone, but the contribution of the mineralocorticoid to the benefit observed in this trial is unknown. Given that hydrocortisone has some mineralocorticoid activity and absolute adrenal insufficiency is rare in sepsis, and using hydrocortisone by itself has been found to be beneficial, the current guidelines for the treatment of septic shock do not advocate use of fludrocortisone[40]

### Role of steroid therapy in shock reversal

Multiple randomised trials in patients with septic shock confirm that low-dose steroid therapy in these patients improves blood pressure, thereby, causing reduction in vasopressor support.[41,42] The postulated mechanisms of the corticosteroid affecting the vascular tone are numerous and cover signal transduction, prostaglandin metabolism, Na+ and Ca+ transport, modulation of adreno-angiotensin, endothelin, mineralocorticoid receptors and inhibition of nitric oxide formation.[43,44] In a French multiple-center, a randomised, controlled trial[45] in 300 patients with severe volume and catecholamine refractory septic shock, low-dose corticosteroid therapy improved survival. This study demonstrated that a low dose of hydrocortisone reduced the risk of death in septic shock patients with relative adrenal insufficiency.

### Duration of corticosteroid therapy

Intravenous hydrocortisone (200–300 mg/day) for seven days in three-to-four divided doses or continuous infusion is recommended in patients with septic shock, who, despite adequate fluid and vasopressor therapy, are not able to maintain their blood pressure. Minneci and colleagues performed a meta-analysis and found that trials done after 1997, used a median total hydrocortisone dose of 1,209 mg versus 23,975 mg in the earlier trials. The later trials also used a steroid taper.[46] Gradual tapering of steroid therapy is required because abrupt discontinuation may lead to rebound hypertension and increase in inflammatory response.[47]

### Adverse effects of corticosteroid therapy

- Worsening of infection that initiated sepsis
- Development of super-infections
- Hypernatraemia
- Hyperglycaemia
- Gastrointestinal bleeding

However, most of these side effects are seen with high-dose corticosteroid therapy. Annane and colleagues performed a meta-analysis on steroid therapy. In data collected from 12 trials, in 1705, a patient’s risk of superinfections in the corticosteroid group was not increased; 1321 patients, in 10 trials did not show increased incidence of gastrointestinal bleeding. In 608 patients from six trials the incidence of hyperglycaemia was not increased.[48]

Results from these data clearly indicate low-dose steroid therapy is safe in septic shock patients.

### Studies on low-dose corticosteroid therapy

The debate on low-dose corticosteroid therapy began in the year 2000, with the publication of the study of Annane and others. They studied 189 patients with septic shock and found that patients having baseline plasma cortisol in the level of ≤9 mg/dl, with corticotrophin stimulation, had the best survival rates.[49]

Another randomised, placebo-controlled, double-blinded trial conducted by Annane and others, in 2002, re-defined the use of low-dose corticosteroid therapy in septic shock patients. A total of 299 patients received either hydrocortisone (50 mg intravenously every six hours) and fludrocortisone (50 mcg tablet once a day) or matching placebos for seven days. Prior to drug administration, the patients received a corticotrophin stimulation test, with two-thirds qualifying as non-responders and the rest as responders. The significant 28-day survival benefit was reported in all patients with corticosteroid responders. The significant 28-day survival benefit was reported in all patients with corticosteroid treatment. A significant improvement in survival was seen primarily in nonresponders.[50]

The next major study to address this issue was the CORTICUS trial. This study was a randomised, double-blinded, placebo-controlled trial that studied 251 septic shock patients who received 50 mg of intravenous hydrocortisone and 248 septic shock patients who received placebo every six hours, for five days. There was no significant survival benefit in the patients treated with hydrocortisone in comparison to the patients treated with placebo (*P*=0.69).[51]

A recent updated meta-analysis published in 2009, indicated the beneficial effect of corticosteroid in septic shock patients, but investigators felt the need for few more research studies in this population of patients.[52]

Annane and co-workers had published an updated review of their research in the past and concluded that regardless of the dose and duration, the use of corticosteroid was not beneficial in septic shock patients.[53]

In the year 2008, the most recent Sepsis Survival Campaign Guidelines have downgraded the role of hydrocortisone in the treatment of septic shock to lower the 2C recommendation, and recommended the use of corticosteroid only to those adults whose blood pressure is not responding to adequate fluid management and vasopressor therapy. The SSC guidelines suggest that patients with septic shock must not receive dexamethasone if hydrocortisone is available (grade 2B). Fludrocortisone is considered optional if hydrocortisone is used (grade 2C). In addition they have also discouraged the corticotrophin stimulation test, to identify septic shock patients requiring steroid therapy with a low-grade 2B recommendation.[54]

## CONCLUSION

In this era of modern anaesthesia, sepsis remains a challenging and complex disease, despite the advances in conventional critical care. Conventional therapies used in the management of sepsis are not up to the mark, therefore, the search continues for newer modalities. Activated protein C was introduced with new hopes in sepsis management. Although APC has proven its worth in various studies conducted previously, data from recent studies have put a question mark on its efficacy. On the other hand the current use of low-dose corticosteroid therapy is somewhat ambiguous, as it has produced its effect mainly in patients who have developed adrenocortical insufficiency. As per the past studies and recommendations the following can be offered:

- Long duration low-dose hydrocortisone therapy, tapered over a period of at least three to five days, is recommended for septic shock patients not responding to adequate fluid and vasopressor therapy.
- Adult patients with sepsis-induced organ dysfunction associated with a clinical assessment of high risk of death (APACHE II≥25 or multiple organ failure) should receive rhAPC if there are no contraindications.
- Adult patients with severe sepsis and low risk of death (APACHE II<20 or one organ failure) should not be given rhAPC.
- The effects in patients with more than one organ failure, but APACHE II<25, are unclear, and in these circumstance one may use the clinical assessment of the risk of death and the number of organ failures, to support the decision.
